# Management of Echocardiography Requests for the Detection and Follow‐Up of Heart Valve Disease: A Consensus Statement From the British Heart Valve Society

**DOI:** 10.1002/clc.70099

**Published:** 2025-02-18

**Authors:** John Chambers, Benoy N. Shah, Madalina Garbi, Brian Campbell, Vassilios S. Vassiliou, Dominik Schlosshan

**Affiliations:** ^1^ British Heart Valve Society London UK; ^2^ Guy's and St Thomas' Hospitals London UK; ^3^ University Hospital Southampton NHS Foundation Trust Southampton UK; ^4^ Royal Papworth Hospital Cambridge Biomedical Campus Cambridge UK; ^5^ Norfolk and Norwich NHS Trust and Norwich Medical School University of East Anglia Norwich UK; ^6^ Leeds NHS Trust Leeds UK

**Keywords:** echocardiography, heart valve, valve lesion

## Abstract

**Background:**

In the aftermath of the Covid19 pandemic and lockdowns, there has been a growing population awaiting transthoracic echocardiograms for potential valvular heart disease. Conducting comprehensive echocardiograms for all individuals may no longer be practical, leading to substantial delays in obtaining the necessary scans. This paper explores an alternative approach, suggesting the consideration of dedicated and shorter scans specifically for patients suspected of having valvular heart disease.

**Hypothesis:**

To address the increasing waiting times and improve heart valve disease detection, the British Heart Valve Society recommends a tiered approach to echocardiograms.

**Methods:**

This approach includes basic/level 1, focused, minimum standard, and disease‐specific scans. Urgency recommendations vary, with individuals experiencing exertional chest pain or pre‐syncope requiring prompt scanning within 2 weeks, ideally at a valve clinic.

**Results:**

Patients without known valve disease but with a murmur and stable breathlessness should be scanned as soon as possible, within a maximum of 6 weeks, balancing local demand and capacity. For those with an asymptomatic murmur and no prior scan, a basic/level 1 study is recommended to triage the necessity for a minimum standard study. Emphasizing appropriate triage for all requests, the statement guides decisions on the necessity for echocardiography, urgency level, and the required scan type.

**Conclusion:**

This practical Consensus Statement from the British Heart Valve Society aims to support appropriate shorter transthoracic echocardiography for patients referred for suspected valvular heart disease. The goal is to enhance capacity in a secure manner, thereby minimizing the risks associated with delays in obtaining timely scans.

## Graphical abstract

Graphical Abstract created using BioRender software.

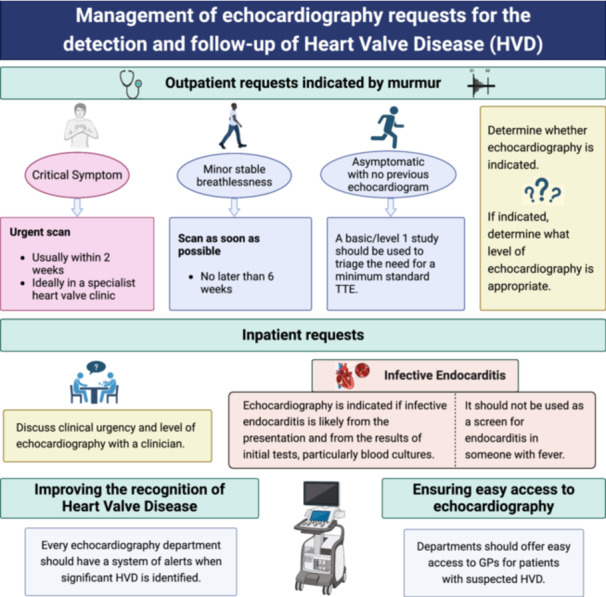

## 1 Introduction

**Figure 1:**
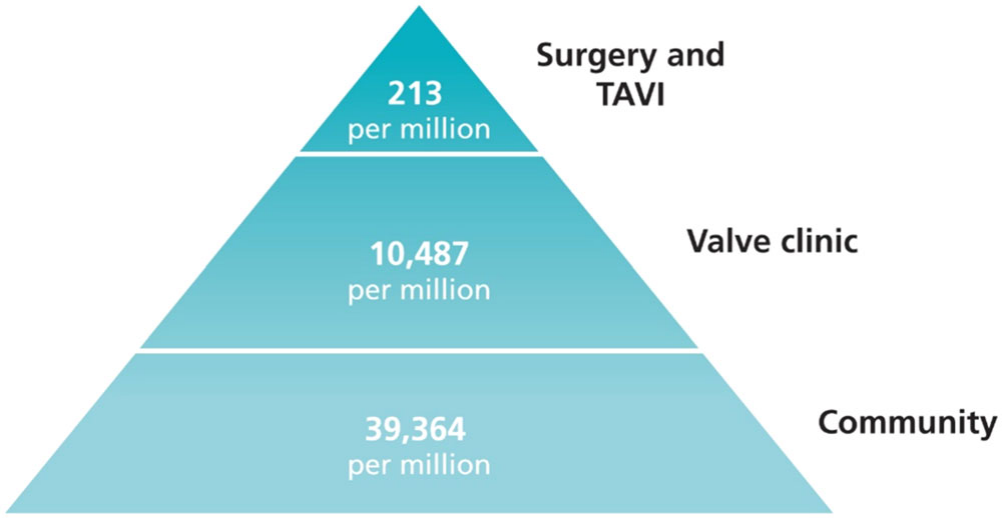
Pyramid of valve disease All numbers are from England and Wales in 2020/21. Tip of the pyramid: There were 4178 aortic valve replacements, 6730 TAVI and 1813 mitral procedures (1118 repairs and 695 replacements) [3, 4]. Valve clinic: From OxVALVE [1] 625 000 people aged > 65 were projected to have moderate or severe valve disease detected. Community prevalence: 1 750 000 people aged > 65 were projected to have detected or undetected moderate or severe HVD [1] and to this was added 1% of the population or 596 000 [2] as a conservative estimate of younger patients with bicuspid valves and mitral prolapse. On census day 2021 the population of England and Wales was 59 597 542 [2].

Heart valve disease (HVD) is a common, but frequently undetected condition [1, 2, 3, 4, 5] (Figure 1). Under‐detection leads to late presentation with almost half of patients having severe (grade III/IV) symptoms at the time of assessment for surgery [6], something that adversely affects their longer‐term prognosis. Moderate or severe HVD is also common in patients with acute de novo heart failure [7].

A major barrier to early detection in the United Kingdom currently, is the waiting time for outpatient transthoracic echocardiography (TTE). Early in 2022, 155 000 people were waiting for TTE [8]. We know that outpatient TTEs are indicated for a murmur in 30%–59% of cases [9, 10, 11] and that about 18% [11] have moderate or severe HVD. This means that up to 91 450 on the waiting list were indicated as a result of clinical auscultation of a murmur and of those. From them, an estimate of 16 000 having moderate or severe HVD. Inevidably, these patients with moderate or severe HVD are at a potential risk of decompensation. In our experience, it is usually extremely difficult, if not impossible, to differentiate those with significant HVD from those with mild or no HVD based on the request form alone, acknowledging that the request can come from a variety of specialists including cardiologists, other physicians, surgeons and general practitioners.

Meanwhile, staffing levels remain inadequate to cope with demand. A pertinent survey by the British Society of Echocardiography [8] in February 2022 noted that 10% of the workforce were locums with up to 3 posts being simultaneously unfilled in some Trusts [8]. This causes significant problems in trying to reduce the waiting lists, with many places seeing an increase in the numbers of patients waiting for echocardiography.

### 1.1 Solutions Incorporating Basic/Level 1 Studies and Focussed Studies

This demand/activity imbalance in the face of inadequate staffing levels requires a rethink of attitudes to echocardiography, to enable more patients to be scanned quicker but maintaining safety.

It would not appear appropriate for every single case to require a full comprehensive study [12] as stipulated by some Imaging Societies. Such comprehensive scan takes a minimum 45 min with the help of a support worker [12]. Whilst the rationale for this approach is to avoid missing pathology in the patients that do receive a scan, it fails to acknowledge the large demand in echocardiography at the moment, and the significant number of patients with a clinically detected murmur, that will not be able to receive their scan for months. Therefore the safety of the patients with a known murmur who are unable to get an echocardiogram for many months or even more than a year, is compromised. As such HVD patients remain without a diagnosis on a waiting list, with potentially adverse outcomes. What transpires, is that in the apparent pursuit of safety for an individual case, the safety of the wider community is compromised. Reassuringly, there is abundant evidence that basic (or level 1) and focussed echocardiograms are safe and sufficient to answer most clinical questions when performed by appropriately trained and experienced specialists. Minimum standard and disease‐specific studies can then be done only when clinically indicated (Table 1).

**Table 1:** The four levels of echocardiogram (TTE).

**Basic/level 1‐**This is effectively an extension of the clinical examination and can be performed with a hand‐held device with color Doppler or a higher‐end machine by an accredited and highly experienced echocardiographer. It typically takes up to 20 min and is used:
To detect pathology requiring immediate correction in the emergency setting (often performed by the physician in charge of the case)
To detect nonacute pathology [13, 14, 15, 16, 17, 18] and determine what further investigations are needed including further echocardiography.
To exclude the need for a minimum standard study in a patient at low clinical risk of disease for example, asymptomatic murmur [14, 16, 17, 18]
**Focussed**‐Typically performed using a mid‐range machine although hand‐held devices with spectral Doppler are now available for example, Clarius, Butterfly, General Electric. This is a basic/level 1 study with additional views directed by the clinical question (e.g., LV measurements to calculate LV mass in hypertension or tricuspid regurgitant Vmax in connective tissues diseases, or transaortic Vmax if the aortic valve is thickened) and must be performed by an accredited and highly experienced echocardiographer with scientific or medical training. It typically takes just over 20 min and is used:
To identify specific abnormalities in screening projects for example, HVD [14, 15, 16, 17, 18, 19]
To detect significant change requiring a comprehensive study in patients with previous minimum standard studies for example, moderate valve disease in a specialist valve clinic
To answer clinically directed focussed questions
**Minimum standard**‐Performed with at least a mid‐range machine by an accredited and experienced echocardiographer. It typically takes up to 45 min.
This is the set of views and measurements without which a study cannot be relied on to exclude significant pathology. It is needed if the basic/level 1 study suggests HVD or other pathology and can be performed as a “bolt on” to basic or focussed studies
**Disease specific**‐Performed using a high‐end machine by an accredited and highly experienced echocardiographer. It can take up to 60 min.
This is a minimum standard study with additional disease specific measurements for known or newly diagnosed HVD

In the early literature, now 15–20 years old, comparisons of basic/level 1 studies using a hand‐held machine against standard TTE on a high‐end machine occasionally showed that abnormalities may be missed [20]. However even with early technology the negative predictive accuracy was 90%–97% [21].

Since then, machines and practice have advanced. The American Society of Echocardiography published guidelines covering indications, practice and training in basic/level 1 and focussed studies in 2013 [22]. The European Society published guidance in 2011 [16] with an update in 2019 [17] stressing that hand‐held devices were inevitable and desirable, mainly based on the improved quality of such hand‐held devices.

More recent clinical studies have used defined protocols for a basic/level 1 study with qualified and appropriately experienced personnel and a hierarchical approach progressing to a broader study (Table 1) guided by abnormalities in the basic/level 1 study. In these clinical validation papers, significant pathology has not been missed [15]. A recent report [23] showed that a basic/level 1 study could be supplemented by focussed “add‐ons” according to the clinical question with no safety concerns and significant saving of time. As an example supporting this, in a chest pain clinic, basic/level 1 studies detected unexpected HVD with a median scan time of 7 min [24]. The adoption of a basic/level 1 protocol in place of a comprehensive study cut the waiting time for an outpatient echocardiogram from 42 to 14 days and a recent meta‐analysis also confirmed the safety and efficacy of hand‐held scans for assessing LV function [25].

**Figure 2:**
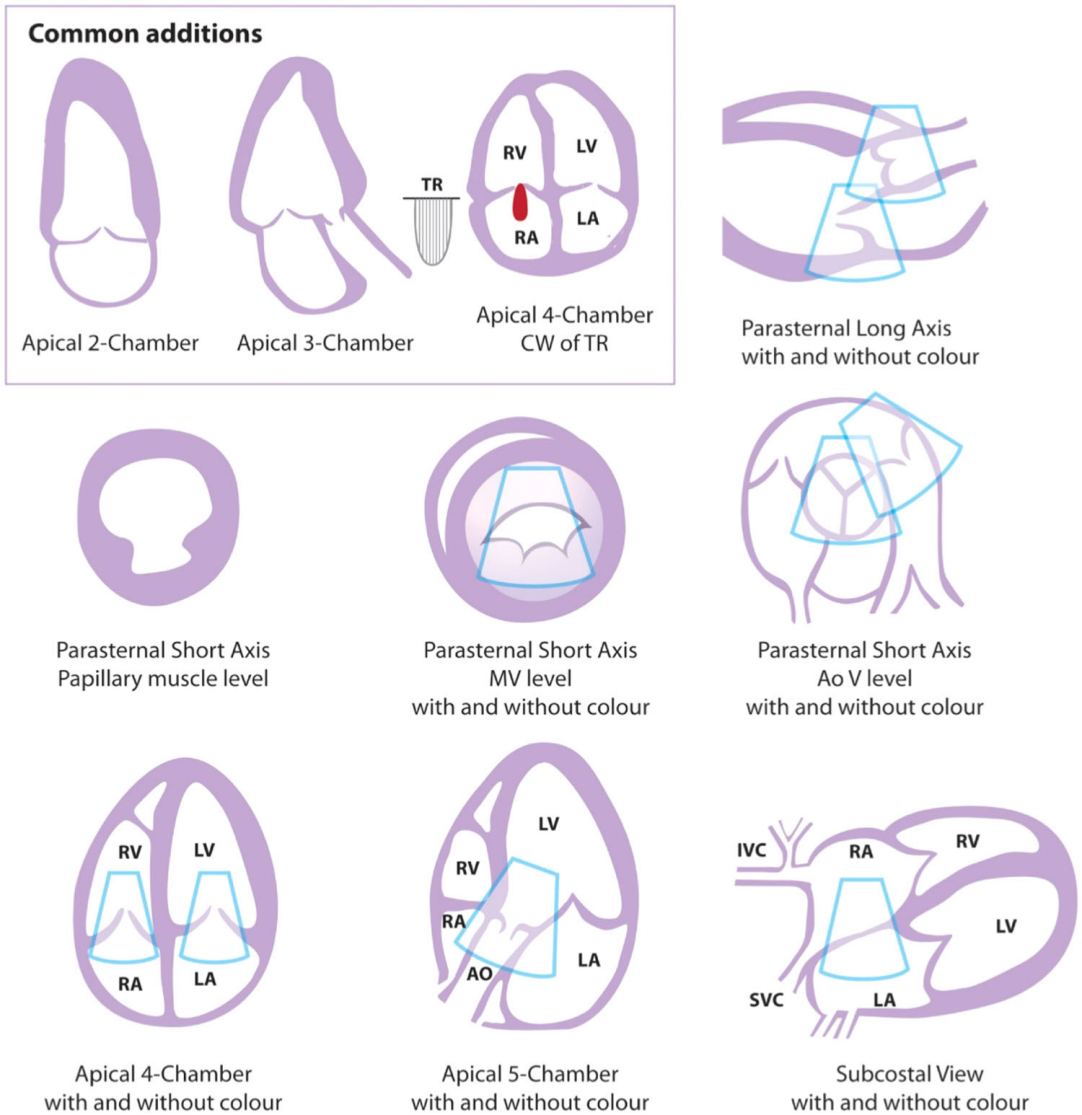
An aide‐memoire showing views for a basic/level 1 echocardiogram. Reproduced with permission from [26].

A suggested aide‐memoire for a basic/level 1 study is given in Figure 2 but individual laboratories may add extra views or measurements as routine for example, apical 2‐chamber view, measurement of LV septal thickness, TR Vmax if tricuspid regurgitation is detected or LA diameter in an electrophysiology request such as for example before consideration of electrical cardioversion or ablation. This underlines that the boundary between a basic/level 1 and a focussed study is porous and can be set by individual departments according to the echocardiography request.

Furthermore, patterns of working have evolved in the 20 years since basic/level 1 studies were introduced. Technicians have become physiologists and increasingly clinical scientists, and the demands of Modernizing Scientific Careers mean that echocardiographers are expected to be able to modify the type of study performed according to the clinical question and any ongoing clinical and technical findings. Different disciplines do not work in silos but in teams, and the cardiologist can discuss or direct the echocardiographer in the type of study required. This is particularly appropriate in a valve clinic but also applies in general lists [23].

This BHVS statement lists a set of recommendations to improve the demand/capacity imbalance for both waiting lists and new requests using these four types of TTE. We acknowledge that in the current climate of an increasing number of individuals on the echocardiography waiting lists and the deficit in the availability of an adequate number of physiologists, it is unrealistic to expect the provision of a state‐of‐the‐art full echocardiographic assessment in everyone with possible HVD. Therefore our recommendations aim to assist with supporting shorter scans (basic/level 1 or focussed) for the majority of individuals, with the possibility of “bolt on” additional images where pathology requiring this is identified.

BHVS Recommendations‐see Graphical Abstract online.

### 1.2 Outpatient Requests Indicated by Murmur

**Figure 3:**
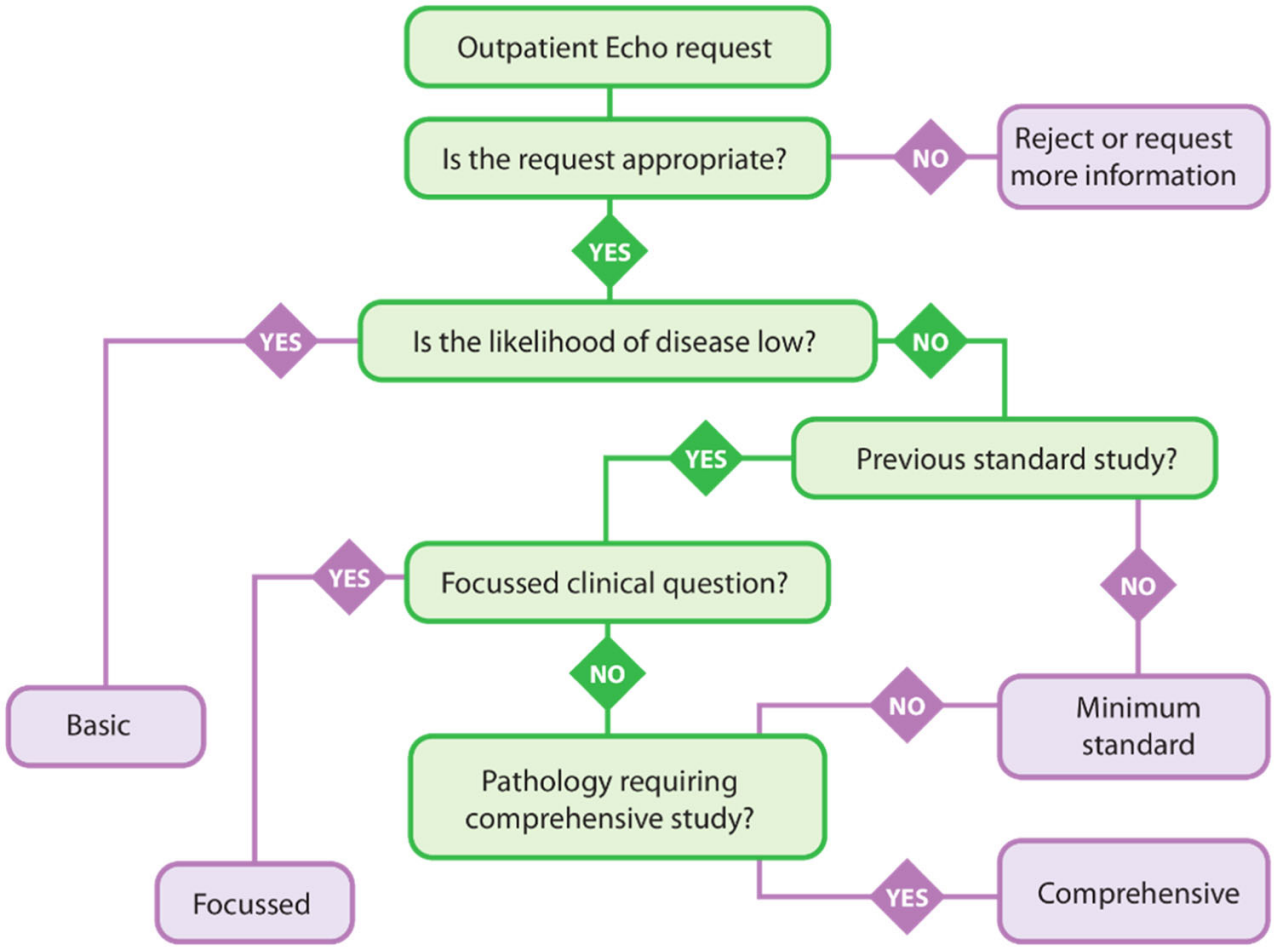
Choosing the level of echocardiogram. Reproduced with permission from [26].

New requests need to be appropriately triaged before being booked for echocardiography (Figure 3). Furthermore, the forms of patients on the outpatient waiting list may need to be reviewed and be reprioritised if necessary. The aim of the triage should be to identify whether an echocardiogram is indeed justified, and if so, the urgency with which this needs to be undertaken and what level of study is needed. Furthermore, administrative support in identifying and printing older echo reports, if not available electronically, would be recommended.

### 1.3 Recommendations Made by the British Heart Valve Society

#### 1.3.1 Recommendation 1

Patients with a murmur and a critical symptom (e.g., exertional chest pain or pre‐syncope or new or severe breathlessness) should be scanned urgently, usually within 2 weeks, and ideally in a specialist heart valve clinic.

##### 1.3.1.1 Background to Recommendation 1

The risk of death or LV dysfunction rises sharply in all HVD after the onset of symptoms. This is particularly the case for severe aortic stenosis in which the risk of sudden death in patients with no symptoms is < 1% per annum but this rises to 3%–4% in the first 3 months after the onset of symptoms and may be as high as 11% at 6 months on a surgical waiting list [27].

This recommendation does not include patients with a murmur and syncope, who should be evaluated as an emergency the same day at either an Emergency Department or a Cardiology setting.

#### 1.3.2 Recommendation 2

Patients without known valve disease with a murmur and minor stable breathlessness should be scanned as soon as possible depending on local demand/capacity, but no later than 6 weeks.

##### 1.3.2.1 Background to Recommendation 2

Breathlessness is a nonspecific symptom often caused by non‐HVD conditions such as obesity or lung pathology. However, it may also indicate HVD. It places the patient below those with a critical symptom but above those with an asymptomatic murmur in terms of the urgency of echocardiography.

#### 1.3.3 Recommendation 3

Requests in asymptomatic murmur without a previous echocardiogram, a basic/level 1 study should be used to triage the need for a minimum standard TTE.

##### 1.3.3.1 Background to Recommendation 3

In TTE indicated for a murmur, the yield of moderate or severe HVD is 11%–18% [9, 28]. Over half have no disease and many do not even have a murmur when auscultated at a specialist murmur clinic [28].

Differentiating a flow murmur from a pathological murmur from the request alone is usually impossible, since the required characteristics for example, soft second heart sound are not included. Furthermore, the accuracy of auscultation skills of clinicians is declining [29] therefore making the original description of the murmur less reliable.

It is therefore inappropriate use of resources to perform comprehensive studies in all patients referred with a diagnosis of a murmur as the majority will have no valve disease.

The basic/level 1 study needs to be performed by a fully accredited and experienced clinical scientist or physiologist who can recognize signs of mild disease requiring a minimum standard TTE (Table 1).

One model, as used at Guy's and St Thomas' Hospitals, London, is a murmur clinic [28] but requests can also be included in a general basic/level 1 list [15, 23]. An alternative model is a focussed study with “add‐ons“ of a pulmonary valve view and a color map of the ventricular septum in the 4‐chamber view.

Further time saving can be arranged locally using health care assistants to prepare patients for all scans including basic/level 1 studies, an approach we highly recommend as it allows the physiologist to focus on the echo studies, be more efficient and is cost‐effective.

#### 1.3.4 Recommendation 4

Determine whether echocardiography is indicated or not. If indicated, determine what level of echocardiography is appropriate for the clinical question (Figure 3).

##### 1.3.4.1 Background to Recommendation 4

Triage requires collaboration between the clinician and echocardiographer. The decision is based on:

- The likelihood of disease (e.g., lower in asymptomatic young patient with murmur).
- Symptoms.
- The results of previous studies.
- The clinical question.

###### 1.3.4.1.1 Examples of Indications Suitable for an Initial Basic/Level 1 Study

- Asymptomatic murmur. These can be assigned to a murmur clinic [28] or a general outpatient basic/level 1 echocardiography session [15].

###### 1.3.4.1.2 Examples of Indications Suitable for a Focussed Study

The cardiologist has a specific clinical question that can be answered by a focussed scan usually in the context of a specialist valve clinic where the clinical assessment and echocardiogram are done at the same time. These are therefore unlikely to appear on a general outpatient list:

- In an asymptomatic patient to look for thickening of a biological aortic valve replacement.
- In a patient with asymptomatic severe aortic stenosis or moderate or severe mitral regurgitation to detect a fall in LV ejection fraction or a rise in the peak velocity of tricuspid regurgitation.
- Previous mild HVD to detect progression requiring a minimum standard study.

###### 1.3.4.1.3 Examples of Indications Requiring a Minimum Standard Study

- Previous studies show significant HVD, and the request is at a guideline‐compliant frequency or otherwise clinically justified.

###### 1.3.4.1.4 Examples of Studies Not Indicated are

- Repeat studies for any HVD earlier than recommended by guidelines in the absence of a clinical change.
- Repeat studies for normally functioning biological valves (without any clinical symptoms/signs to suggest failure) before guideline indications [30].
- Repeat study for most patients with mild native valve regurgitation and for studies previously shown to be normal.
- Repeat studies in the absence of symptoms/signs, for patients who received TAVI, who would not be suitable for re‐intervention.

### 1.4 Inpatient Requests

#### 1.4.1 Recommendation 5

Discuss clinical urgency and level of echocardiography with a clinician.

##### 1.4.1.1 Background to Recommendation 5

The need for clinical collaboration is greater for inpatient than for outpatient requests since the presentation may be acute, not necessarily associated with a murmur and the dangers of progression much higher.

There can be no safe target time‐delays and every case must be considered individually.

No level of echocardiography is universally applicable. A combination of a basic/level 1 study can be used to exclude life‐threatening pathology and a minimum standard study performed at greater leisure if required.

###### 1.4.1.1.1 Examples of Indications Suitable for an Initial Basic/Level 1 Study

- Incidental murmur in a patient admitted with a nonvalve problem for example, neck of femur fracture. It is reasonable to perform a basic/level 1 study in patients with a murmur as an emergency if identifying HVD will change management.

- After insertion of an electrical device or other invasive intervention a basic/level 1 study is frequently sufficient to exclude a new pericardial effusion.
- In an acutely unwell patient, a basic/level 1 study may indicate the need for immediate life‐saving treatment.

###### 1.4.1.1.2 Examples of Indications Suitable for a Focussed Study

- Predischarge after cardiac surgery to detect pathology that might require immediate management for example, large pericardial effusion or LV dysfunction or prosthetic valve dysfunction.

###### 1.4.1.1.3 Examples of Indications Requiring a Minimum Standard Study

- Patients with known HVD admitted with heart failure.
- Murmur after acute myocardial infarction since it may indicate mitral regurgitation or a VSD.
- Urgent echocardiography should be done if there is unexplained LV failure or shock since these might complicate critical HVD.

###### 1.4.1.1.4 Examples of Studies Often Not Indicated

- Fever with a low clinical likelihood of infective endocarditis (see recommendation 6).
- Recent TTE with no evidence of clinical deterioration.

#### 1.4.2 Recommendation 6

Echocardiography is indicated if infective endocarditis is likely from the presentation and from the results of initial tests, particularly blood cultures. It should not be used as a screen for endocarditis in someone with fever.

##### 1.4.2.1 Background to Recommendation 6

Requests based on presence of fever alone are common, but not indicated. There is a trend to use the Duke criteria as a protocol for tests. The Duke criteria are intended to aid the formulation in clinical practice. The initial suspicion of infective endocarditis is based on clinical characteristics and not presence of fever alone. We would advocate for a multidisciplinary approach involving medical teams, cardiology and infectious disease/microbiology before requesting echocardiography.

Using echocardiography as a “fever screen” wastes resources since the yield is very low [31] and risks minor abnormalities or normal variants confounding the diagnosis for example, minor calcification, fibrin strands.

Early antibiotic therapy is key and particularly important for reducing the risk of embolization. Therefore, patients with a moderate or high likelihood of infective endocarditis should have echocardiography on the day of the request. If the patient is in heart failure or critically ill and might require urgent or emergency surgery, the study should be performed immediately. Ideally a member of the infective endocarditis team will guide the clinical urgency.

A comprehensive study (and not a basic/level 1 or focussed study) is required since multiple views may be needed to show vegetations and local complications including an aortic root abscess, and communication with the cardiology team for consideration of transoesophageal echocardiography.

### 1.5 Improving the Recognition of HVD

#### 1.5.1 Recommendation 7

Every echocardiography department should have a system of alerts when significant HVD is identified.

##### 1.5.1.1 Background to Recommendation 7

There is anecdotal evidence that the presence of HVD may not be communicated to an appropriate clinician. The department needs to have a system for reporting significant HVD to the clinical scientist/physiologist running the department, the referring clinician or a supervising cardiologist, preferably the cardiologist in charge of the valve clinic.

There could be a lack in general practitioner (GP) training to facilitate full understanding of echocardiography reports [32]. It is therefore of particular importance that open access studies requested by the GP showing HVD are discussed with a cardiologist to write recommendations for management. Some centers already arrange an appointment to a valve clinic automatically, if clinically appropriate, which cuts out delays between the hospital and GP, avoids risk of the referral not happening and is popular with GPs. If possible, this would be the recommended approach.

### 1.6 Ensuring Easy Access to Echocardiography

#### 1.6.1 Recommendation 8

Departments should offer easy access to GPs for patients with suspected HVD.

##### 1.6.1.1 Background to Recommendation 8

The usual method of referral is within an open access service. Only 65 (76%) of departments offered this in 2022 [8] and more should be encouraged to start.

Ways of extending access locally have been explored in the past for example, hand‐held devices in a GP practice [19] but these have not been successful yet, therefore would not be recommended.

Atrial fibrillation is a recognized indication for TTE since HVD is more likely in the presence of atrial fibrillation than sinus rhythm but also needed to assess systolic size and function [1]. It is imperative that detection of new Atrial Fibrillation on a scan is relayed to the referring clinician, or GP and the patient informed so as to ensure that assessment for thromboembolic risk and consideration of anticoagulation happens. A standard leaflet for Atrial Fibrillation (such as the British Heart Foundation leaflet) [33] could be given to the patients.

### 1.7 Expected Benefits of These Recommendations

The expectation is that utilizing shorter scans will enable a substantially greater number of individuals on the waiting list to undergo echocardiography within a specific time frame. This approach is anticipated to be safe, given that most do not have significant valvular disease and the option to extend the scans if an abnormality is identified exists. Consequently, it mitigates the risk of patients with significant valvular heart disease enduring prolonged waits for their minimum standard scan. While there may be a small subset of individuals with nonvalvular pathology that could be overlooked in a basic/level 1 or focused scan, the overall safety benefits at a population level outweighs this potential limitation.

## 2 Conclusion

This Consensus Statement from the British Heart Valve Society aims to address the increasing waiting lists for echocardiography for patients with suspected valvular heart disease in the UK. It may not be applicable for other countries where echocardiography provision could vary. While a full comprehensive study has long been considered necessary, currently we advocate for shorter studies to enable a higher number of patients to have a suitable scan earlier on. The objective of our Recommendations is to increase capacity safely, thus minimizing potential risks linked to delays in obtaining timely scans.

## Author Contributions

All authors are Council Members of the British Heart Valve Society. All authors discussed and approved the recommendations. Prof John Chambers wrote the first draft. All authors contributed in revising the original draft. Prof Chambers passed away 3 days before submission of this manuscript.

## Conflicts of Interest

The authors declare no conflicts of interest.

## Data Availability

This manuscript present a Consensus Statement from the British Heart Valve Society. No new data has been presented.
